# Phenotypic Profile of *Mycobacterium tuberculosis*-Specific CD4 T-Cell Responses in People With Advanced Human Immunodeficiency Virus Who Develop Tuberculosis-Associated Immune Reconstitution Inflammatory Syndrome

**DOI:** 10.1093/ofid/ofac546

**Published:** 2022-10-17

**Authors:** Raymond M Moseki, Daniel L Barber, Elsa Du Bruyn, Muki Shey, Helen Van der Plas, Robert J Wilkinson, Graeme Meintjes, Catherine Riou

**Affiliations:** Wellcome Center for Infectious Diseases Research in Africa (CIDRI-Africa), University of Cape Town, Cape Town, South Africa; Institute of Infectious Disease and Molecular Medicine (IDM), University of Cape Town, Cape Town, South Africa; T Lymphocyte Biology Section, Laboratory of Parasitic Diseases, National Institute of Health, National Institute of Allergy and Infectious Diseases, Bethesda, Maryland, USA; Wellcome Center for Infectious Diseases Research in Africa (CIDRI-Africa), University of Cape Town, Cape Town, South Africa; Institute of Infectious Disease and Molecular Medicine (IDM), University of Cape Town, Cape Town, South Africa; Wellcome Center for Infectious Diseases Research in Africa (CIDRI-Africa), University of Cape Town, Cape Town, South Africa; Department of Medicine, University of Cape Town, Cape Town, South Africa; Institute of Infectious Disease and Molecular Medicine (IDM), University of Cape Town, Cape Town, South Africa; Department of Medicine, University of Cape Town, Cape Town, South Africa; Wellcome Center for Infectious Diseases Research in Africa (CIDRI-Africa), University of Cape Town, Cape Town, South Africa; Institute of Infectious Disease and Molecular Medicine (IDM), University of Cape Town, Cape Town, South Africa; Department of Medicine, University of Cape Town, Cape Town, South Africa; Department of Infectious Diseases, Imperial College London, London, United Kingdom; The Francis Crick Institute, London, United Kingdom; Wellcome Center for Infectious Diseases Research in Africa (CIDRI-Africa), University of Cape Town, Cape Town, South Africa; Institute of Infectious Disease and Molecular Medicine (IDM), University of Cape Town, Cape Town, South Africa; Department of Medicine, University of Cape Town, Cape Town, South Africa; Wellcome Center for Infectious Diseases Research in Africa (CIDRI-Africa), University of Cape Town, Cape Town, South Africa; Institute of Infectious Disease and Molecular Medicine (IDM), University of Cape Town, Cape Town, South Africa; Department of Pathology, Division of Medical Virology, University of Cape Town, Cape Town, South Africa

**Keywords:** HIV-1/TB coinfection, immune activation, paradoxical TB-IRIS, Tbet/Eomes, Th-1 responses

## Abstract

**Background:**

Tuberculosis-associated immune reconstitution inflammatory syndrome (TB-IRIS) is a frequent complication of cotreatment for TB and human immunodeficiency virus (HIV)-1. We characterized *Mycobacterium tuberculosis* (Mtb)-specific CD4 T-cell phenotype and transcription factor profile associated with the development of TB-IRIS.

**Methods:**

We examined the role of CD4 T-cell transcription factors in a murine model of mycobacterial IRIS. In humans, we used a longitudinal study design to compare the magnitude of antiretroviral therapy, activation, transcription factor profile, and cytotoxic potential of Mtb-specific CD4 T cells between TB-IRIS (*n* = 25) and appropriate non-IRIS control patients (*n* = 18) using flow cytometry.

**Results:**

In the murine model, CD4 T-cell expression of Eomesodermin (Eomes), but not Tbet, was associated with experimentally induced IRIS. In patients, TB-IRIS onset was associated with the expansion of Mtb-specific IFNγ^+^CD4 T cells (*P* = .039). Patients with TB-IRIS had higher HLA-DR expression (*P* = .016), but no differences in the expression of T-bet or Eomes were observed. At TB-IRIS onset, Eomes^+^Tbet^+^Mtb-specific IFNγ^+^CD4^+^ T cells showed higher expression of granzyme B in patients with TB-IRIS (*P* = .026).

**Conclusions:**

Although the murine model of *Mycobacterium avium* complex-IRIS suggests that Eomes^+^CD4 T cells underly IRIS, TB-IRIS was not associated with Eomes expression in patients. *Mycobacterium tuberculosis*-specific IFNγ^+^CD4 T-cell responses in TB-IRIS patients are differentiated, highly activated, and potentially cytotoxic.

Although antiretroviral therapy (ART) has substantially reduced human immunodeficiency virus (HIV)-1-related morbidity and mortality in people with HIV(PWH) and tuberculosis (TB) [1], TB-immune reconstitution inflammatory syndrome (TB-IRIS) frequently complicates management [2, 3]. Tuberculosis-IRIS has an estimated incidence of 18% across cohorts and an attributable mortality rate of 2% [4].

Two forms of TB-IRIS are recognized: (1) unmasking TB-IRIS, which occurs in patients with undiagnosed TB who present with severe inflammatory features of TB during the first 3 months of ART; and (2) paradoxical TB-IRIS, which occurs in patients started on TB treatment before ART who experience recurrent, new, or worsening symptoms and signs of TB within the first months of initiating ART [5, 6]. The major risk factors for paradoxical TB-IRIS include a low CD4 count before ART initiation, higher HIV-1 viral load, a short interval between TB treatment and ART initiation, and disseminated TB [7, 8].

Innate immune responses including inflammasome activation [9, 10], monocyte and natural killer cell activation [11, 12], neutrophilia [12, 13], and dysregulation of the complement system in monocytes [14] have been associated with TB-IRIS. Elevated concentrations of proinflammatory cytokines [15, 16] and matrix degrading metalloproteinases [17] have been described at TB-IRIS onset. Moreover, monocyte subset frequency and circulating inflammatory mediators can independently predict TB-IRIS disease [18, 19]. Expansion of pathogen-specific CD4^+^ T cells has been observed in association with TB-IRIS [20–23]. Pathogen-specific CD4^+^ T cells from patients with IRIS have been reported to be highly activated [24] and polyfunctional [25]. In a recent study, it was reported that immunosuppressed PWH, who were coinfected with *Mycobacterium avium* complex (MAC) and subsequently developed MAC-IRIS, had higher expression of Eomesodermin (Eomes) compared with Tbet in MAC-specific IFNγ^+^CD4^+^ T cells at the onset of IRIS [26]. Eomesodermin and Tbet are members of the T-box deoxyribonucleic acid binding family of transcription factors with structural similarities and overlapping expression [27]. Eomesodermin is involved in the development of cytotoxic T lymphocyte activity [27], whereas Tbet is a Th-1 lineage-defining transcription factor [28].

Th-1 responses have been implicated in a mouse model of MAC-IRIS [20, 21]. As a result, we capitalized on the existing mouse model of IRIS to investigate phenotypic CD4 T-cell features that may be associated with IRIS in mice and compare these with findings in patients developing TB-IRIS in a prospective cohort study of immunosuppressed PWH and TB initiating ART.

## METHODS

### *Mycobacterium avium*-Immune Reconstitution Inflammatory Syndrome Induction in Mice

C57BL/6J-(knockout [KO]) TCRα mice (6–8 weeks old) were intravenously infected with 1 × 10^6^ colony-forming units of *M avium* (strain SmT 2151). After at least 40 days, CD4 T cells were isolated from C57BL/6, B6.129S6-Tbx21tm1Glm/J mice (The Jackson Laboratory, Bar Harbor, ME) or Eomes^fl/fl^CD4-CRE^+^ uninfected mice using positively selecting microbeads (Miltenyi Biotec, Auburn, CA), and 1 × 10^6^ cells were intravenously transferred into chronically infected T cell-deficient mice. All mice were maintained and bred at National Institute of Allergy and Infectious Diseases (NIAID), National Institutes of Health (Bethesda, MD). All animals were housed at an Association for the Assessment and Accreditation of Laboratory Animal Care-approved facility at the NIAID according to the National Research Council's Guide for the Care and Use of Laboratory Animals.

### Participants in Clinical Study

Samples were obtained from PWH and TB initiating ART enrolled in a prospective observational study conducted at Brooklyn Chest Tuberculosis Hospital between May 2009 and November 2010 in Cape Town, South Africa [29]. All patients were ART naive, and those with rifampicin-resistant TB were excluded. Tuberculosis diagnosis was based on smear, culture, or clinical diagnosis. The first TB episode was treated with standard first-line regimen of rifampicin (R), isoniazid (H), pyrazinamide, and ethambutol for 2 months followed by 4 months of RH regimen. In patients with subsequent episodes, streptomycin was added for 2 months. Tuberculosis-IRIS was diagnosed per International Network for the Study of HIV-associated IRIS criteria [5]. Human immunodeficiency virus-1 treatment included lamivudine and efavirenz with stavudine or tenofovir depending on guidelines at the time.

### Patient Consent Statement

Written informed consent was obtained from all participants. The study design was approved by the Human Research Ethics Committee (HREC REF: 049/2009 and 809/2018) of the University of Cape Town. Clinical and other immunological findings from this cohort have been published [9, 29, 30].

### Peripheral Blood Mononuclear Cell Isolation and Stimulation

Peripheral blood mononuclear cells (PBMCs) were isolated by Ficoll-Hypaque density gradient centrifugation (ALC-PK121R; GE Healthcare), cryopreserved, and stored. Cryopreserved PBMCs were thawed and rested at 37°C in Roswell Park Memorial Institute 1640 medium containing 10% heat-inactivated fetal calf serum for 4 hours before antigen stimulation. Peripheral blood mononuclear cells (2 × 10^6^ cells) were stimulated with a peptide pool constituted of 300 Mtb-derived peptides (Mtb300, 1.5 µg/mL) [31] in the presence of anti-CD28 and anti-CD49d antibodies (both at 1 μg/mL; BD, Franklin Lakes, NJ) and brefeldin-A (10 μg/mL; Sigma, St. Louis, MO) for 6 hours. Unstimulated cells, incubated with costimulatory antibodies and Brefeldin A only, were used as controls.

### Cell Staining and Acquisition

Samples with a cell count of less than 1 million or a viability score of less than 20% were excluded. After stimulation, cells were washed, stained with a viability marker (Live/Dead Fixable Near-InfraRed marker; Invitrogen, Carlsbad, CA) for 10 minutes at room temperature, and subsequently surface stained with the following antibodies: CD4-PerCP-cy5.5, PD1-BV421, HLA-DR-BV605, CD14-Allophycocyanin/H7, CD19-Allophycocyanin/H7 (all from BioLegend, San Diego, CA), and CD8-Alexa700 (BD) for 30 minutes at room temperature. Cells were fixed and permeabilized using the eBioscience Foxp3 fixation buffer for 30 minutes at room temperature and stained with IFNγ-BV711 (BioLegend), TNFα-FITC (BioLegend), granzyme B-BV510 (BD), Eomes-eFluor 660 (e-Bioscience, San Diego, CA), Tbet-PEcy7 (e-Bioscience), and CD3-BV785 (BioLegend) for 45 minutes at 4°C. Cells were washed and resuspended in 1% formaldehyde in phosphate-buffered saline. Samples were acquired in the BD LSRII, and data were analyzed using FlowJo software version 9.9.6 (BD). The gating strategy is presented in Supplementary Figure 1. A positive Mtb-specific IFNγ response was defined as 3-fold higher than the background measured in the presence of costimulatory antibodies without antigen. For the phenotypic analyses of Mtb-specific IFNγ^+^CD4^+^ T cells, only Mtb responses with more than 20 events were considered. Protocols were compliant with the guidelines for flow cytometry in immunological studies [32]. Although we analyzed immunological characteristics of live cells, our cohort included 8 samples with a viability below 50% (median: 67% [range, 96%–22%]). Before assessing immunological phenotypic characteristics of our cohort, we ascertained whether sample viability affected immunological expression of markers (particularly Tbet and Eomes) in our cohort. There was no correlation between sample viability and the expression of Tbet and Eomes at all measured time points (data not shown).

### Statistical Analyses

For analyses, samples from TB-IRIS and non-IRIS groups were classified into 4 categories based on sample timing in relation to ART: baseline (BL) include samples collected within 7 days before or on the day of ART initiation, Week 2 (W2) samples collected between day 1 and 14 on ART, Week 4 (W4) samples collected between day 15 and 30 on ART, Week 6 (W6) samples collected between day 31 and 65 on ART. Paired samples were analysed using the Wilcoxon signed-ranked Student *t* test, whereas the Mann-Whitney *U* test was used to compare unpaired samples for all time points between TB-IRIS and non-IRIS groups. A *P* value of .05 or less was considered statistically significant. All statistical analyses were performed using Prism (v8.0.2; GraphPad Software Inc., San Diego, CA).

## RESULTS

### Role of Eomesodermin and Tbet in CD4 T Cells During Experimentally Induced Immune Reconstitution Inflammatory Syndrome

**Figure 1.**
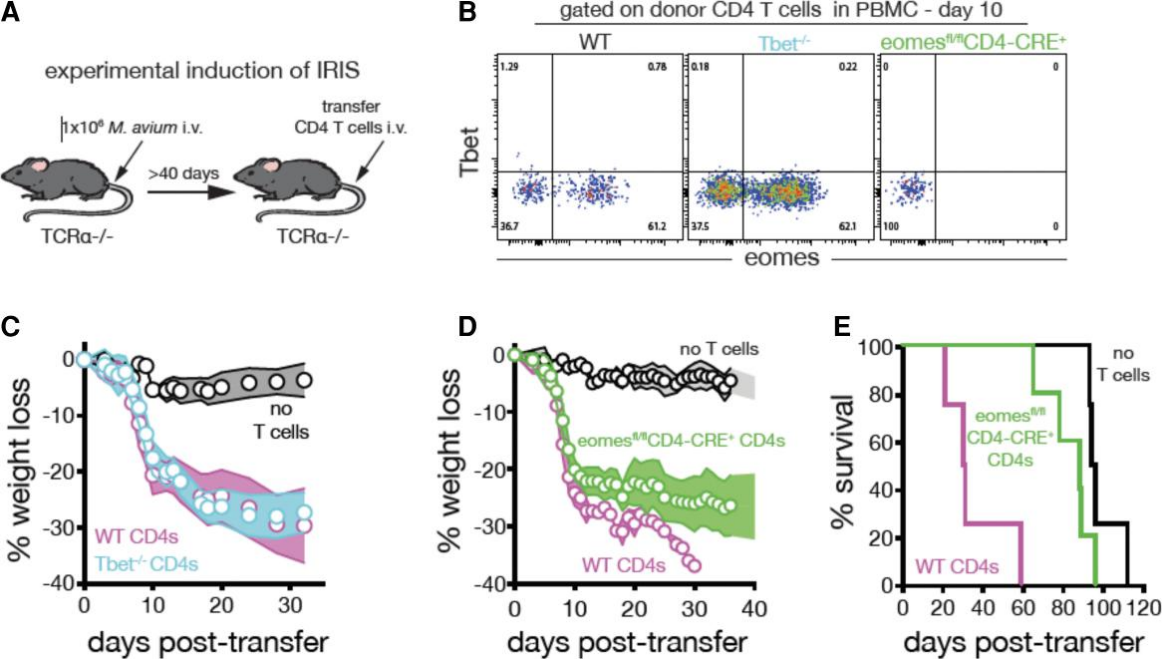
CD4 T-cell expression of Eomesodermin (Eomes) promotes mycobacterial immune reconstitution inflammatory syndrome (IRIS) in a murine model. (*A*) To model IRIS in mice, TCRα^−/−^ mice harboring a chronic *Mycobacterium avium* infection were given injections of purified CD4 T cells from uninfected donor mice. (*B*) *Mycobacterium avium*-infected TCRα^−/−^ mice were given injections of wild-type (WT), Tbet-deficient, or Eomes-deficient CD4 T cells. The donor CD4 T cells (CD4^+^TCRβ^+^CD3^+^) were analyzed in the Peripheral blood mononuclear cells (PBMC) on day 10 postinfection for the expression of Tbet and Eomes (plots are concatenated from *n* = 8 mice/group). (*C*) *Mycobacterium avium*-infected TCRα^−/−^ mice were given injections of either WT or Tbet-deficient CD4 T cells and monitored for weight loss (*n* = 5 mice/group). (*D*) *Mycobacterium avium*-infected TCRα^−/−^ mice were given injections of no T cells, WT, or Eomes-deficient CD4 T cells and monitored for weight loss. (*E*) Survival of mice receiving WT or Eomes-deficient CD4 T cells. *n* = 4 to 5 mice/group. Error bars represent the standard deviation. Data are representative of at least 4 independent experiments each.

To model IRIS, T cell-deficient (TCRα^−/−^) mice were infected with *M avium*. This reproduced a lymphopenic host harboring a mycobacterial infection. After 40–60 days, the mice were given injections of CD4 T cells to mimic the reconstitution of T cells that occurs after ART (Figure 1*A*). To examine the expression of Eomes and Tbet in CD4^+^ T cells and their potential involvement in the murine model of IRIS, we gave mice injections of wild-type (WT), Tbet^−/−^, and Eomes-deficient CD4 T cells and examined the donor CD4 T cells 10 days postinjection. We found that during murine IRIS, CD4 T cells surprisingly expressed little Tbet. Instead, a significant population of Eomes^+^CD4^+^ T cells was observed (Figure 1*B*). Wild-type and Tbet^−/−^CD4^+^ T cells induced similar levels of weight loss (Figure 1*C*). In contrast, recipients of Eomes-deficient CD4^+^ T cells displayed less weight loss and longer survival compared with mice that were given injections of WT CD4^+^ T cells (Figure 1*D*and*E*). We concluded that CD4^+^ T cells utilize Eomes but not Tbet, to drive *M avium* IRIS in this animal model. These findings prompted us to next examine the role of Eomes expressing CD4^+^ T cells in human TB-IRIS.

### Clinical Characteristics of the Cohort

Sufficient samples for immunological analyses were available for 43 inpatients infected with HIV-1 and being treated for TB when starting ART: 25 patients developed TB-IRIS and 18 patients did not (non-IRIS controls). The demographic and clinical characteristics of the 2 groups are summarized in Table 1. In both groups, over three quarters of patients had evidence of extrapulmonary TB and approximately 20% had neurological TB, a common reason for TB patients in South Africa to be admitted to a TB hospital. It is notable that 7 of 25 (28%) of TB-IRIS and 4 of 18 (22%) of non-IRIS patients were on treatment with corticosteroids at the time of starting ART, the most frequent indication being neurological TB. We previously demonstrated that corticosteroid therapy had no significant effect on the frequency of Mtb-specific CD4 T cells [33]. The median CD4 count at the start of ART was lower in TB-IRIS patients (median: 68 cells/mm^3^) compared with non-IRIS patients (median 111 cells/mm^3^) (*P* = .009). The median duration of TB treatment before initiation of ART was similar for the groups (median 37 days in TB-IRIS vs 32 days in non-IRIS patients). The duration of ART before developing TB-IRIS symptoms was a median of 15 days. Additional clinical data are presented in Supplementary Table 1.

**Table 1.** Clinical Characteristics of Patients Who Developed Tuberculosis Immune Reconstitution Inflammatory Syndrome (TB-Iris, *n* = 25) and Those Who Did Not (Non-IRIS, *n* = 18)

Patient Characteristics	TB-IRIS (*n* = 25)	Non-IRIS (*n* = 18)	*P* Value
Age (Median, IQR) (years)	34 (22–52)	33 (24–55)	ns
Female sex (*n*, %)	13 (52%)	12 (66%)	
Previous TB (*n*, %)	15 (60%)	10 (55%)	
TB type (*n*, %)			
PTB	4 (16%)	2 (11%)	
EPTB	4 (16%)	5 (27%)	
EPTB and PTB	17 (68%)	11 (61%)	
TB meningitis/neuro-TB (*n*, %)	7 (23%)	4 (21%)	
TB confirmation (*n*, %)			
Cultured Mtb	9 (36%)	6 (33%)	
Smear	6 (24%)	2 (11%)	
Clinicoradiological	10 (40%)	10 (55%)	
Hb (median, IQR) (g/dL)	9.1 (6.4–13)	9.4 (5.9–14.0)	ns
CD4 nadir (median, IQR)	49 (11–209)	70 (4–272)	ns
CD4 count (cells/mm^3^) at week 0 (median, IQR)	68 (21–521)	111 (4–662)	**.009**
CD4 count (cells/mm^3^) at week 4 (median, IQR)	164 (23–556)	276 (21–514)	ns
Log_10_ HIV VL at week 0 (median, IQR)	5.73 (3.96–7.78)	5.8 (4.21–7.15)	ns
Log_10_ HIV VL at week 4 (median, IQR)	2.72 (0–3.88)	2.68 (1.32–3.3)	ns
Duration TB treatment to ART (median, IQR) (days)	37 (14–99)	32 (13–91)	ns
Duration ART to TB-IRIS (median, IQR) (days)	15 (4–49)	na	-
On steroid treatment at week 0 (*n*, %)	7 (28)	4 (22)	ns

Abbreviations: ART, antiretroviral treatment; EPTB, extrapulmonary tuberculosis; Hb, hemoglobin; HIV, human immunodeficiency virus; IQR, interquartile range; Mtb, *Mycobacterium tuberculosis*; na, not applicable; ns, not significant; PTB, pulmonary tuberculosis; TB, tuberculosis; VL, viral load.

NOTE: The Wilcoxon rank-sum test was used to compare all continuous variables, and the Mann-Whitney test was used to compare categorical variables. The bold font was to highlight the significant *P*-value for the CD4 count measurement in patients with TB-IRIS compared to non-IRIS controls. Notably, lower CD4 counts have been noted to be one of the major predisposing factors to TB-IRIS.

### Expansion of *Mycobacterium tuberculosis*-Specific CD4^+^ T Cells at Tuberculosis-Immune Reconstitution Inflammatory Syndrome Onset

**Figure 2.**
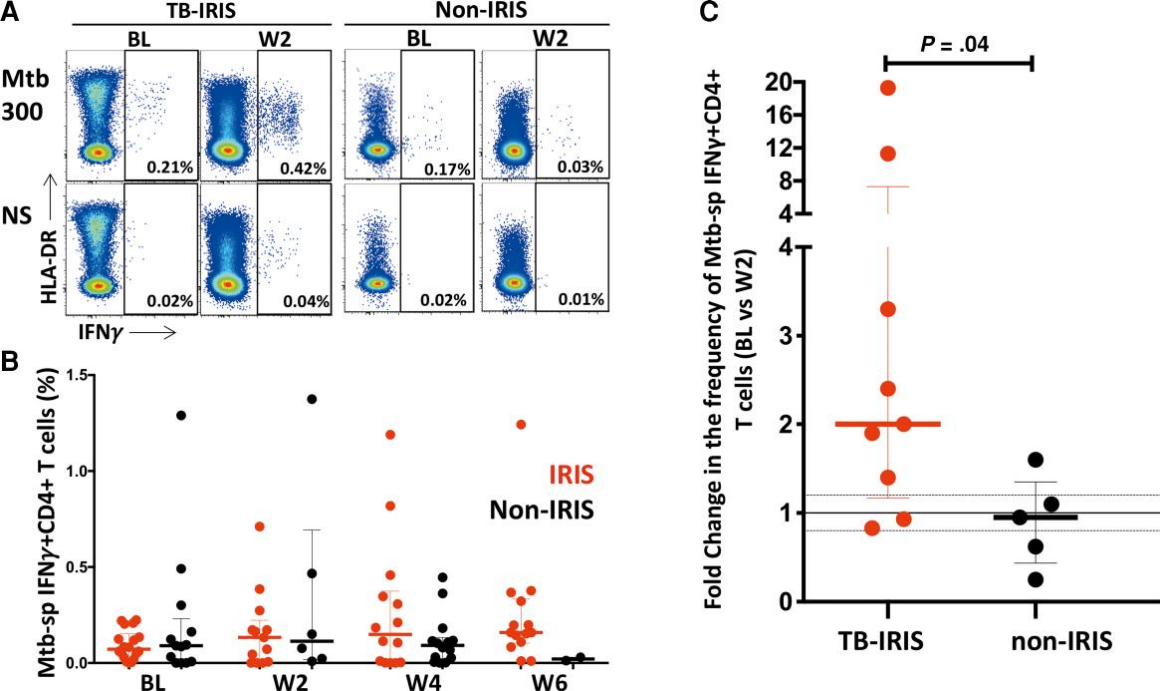
Frequencies of *Mycobacterium tuberculosis* (Mtb)-specific IFNγ^+^CD4^+^ T cells in tuberculosis-immune reconstitution inflammatory syndrome (TB-IRIS) and non-IRIS patients. (*A*) Representative flow plots of IFNγ production in response to Mtb peptide pool (Mtb300) and nonstimulated controls (NS) at baseline ([BL] before initiation of antiretroviral therapy [ART]) and 2 weeks on ART (W2). (*B*) Frequencies of IFNγ-producing CD4^+^ T cells in TB-IRIS (red) from baseline (BL, *n* = 16), through 2 weeks ([W2] *n* = 9), 4 weeks ([W4] *n* = 10) and 6 weeks ([W6] *n* = 12) and non-IRIS (black) from BL = 11, through W2, *n* = 4, W4, *n* = 8 and W6, *n* = 1 on ART. (*C*) Fold change in the frequency of IFNγ^+^CD4^+^ T cells in TB-IRIS and non-IRIS patients between baseline (before ART) and 2 weeks on ART. The Wilcoxon signed-rank test was used for the statistical comparison of paired samples, and the Mann-Whitney *U* test was used for unpaired samples. Only statistically significant data with a *P* value of .05 or less are indicated on graphs.

For phenotypic analyses, we first compared the magnitude of Mtb-specific IFNγ^+^CD4^+^ T-cell responses between the patient groups before the initiation of ART (BL), at WK 2, 4, and 6 on ART. Representative examples of IFNγ production by CD4^+^ T cells after Mtb300 stimulation are presented in Figure 2*A*. We observed no differences in the frequency of Mtb-specific IFNγ^+^CD4^+^ T cells between the 2 groups in cross-sectional comparisons at any time point (Figure 2*B*). However, the fold change in Mtb-specific IFNγ^+^CD4^+^ T-cell frequency between BL and week 2 was significantly higher in the TB-IRIS group compared to the non-IRIS group (median fold change = 1.9 [interquartile range {IQR}, 0.83–19.3] and 0.9 [IQR, 0.25–1.6], respectively; *P* = .04) (Figure 2*C*). This significant increase was exclusively observed in TB-IRIS patients between BL (median: 0.08% [IQR, 0.0–0.2]) and 2 weeks on ART (median: 0.13% [IQR, 0.0–0.71]; *P* = .039) (Supplementary Figure 2). We next investigated the phenotype of Mtb-specific IFNγ^+^CD4^+^ T cell that could potentially characterize the role of these cells in the pathogenesis of TB-IRIS in humans.

### No Differences in the Expression of Eomesodermin or Tbet Between Patients With and Without Tuberculosis-Immune Reconstitution Inflammatory Syndrome at Any Tested Time Point

Based on our mouse model data and a recent report by Hsu et al [26] reporting that Eomes was significantly upregulated over Tbet in MAC-specific IFNγ^+^CD4 T cells of MAC-IRIS patients at disease onset, we determined whether these transcription factors were differentially expressed between TB-IRIS and non-IRIS patients. We observed no differences in the frequency of Mtb-specific IFNγ^+^Eomes ^+^ CD4^+^ T cells between the 2 clinical groups at BL or any time point on ART (Supplementary Figure 3).

Eomesodermin and Tbet expression in Mtb-specific IFNγ^+^CD4 T cells were highly variable between patients but not statistically different between the 2 groups at baseline (Supplementary Figure 4) or any other time point (data not shown). The expression of Eomes in Mtb-specific IFNγ^+^CD4 T cells at baseline was approximately 50% and was comparable between the 2 groups (Supplementary Figure 4*B*). Likewise, Tbet expression in Mtb-specific IFNγ^+^CD4^+^ T cells was comparable between TB-IRIS and non-IRIS groups; approximately 60% of cells expressed intermediate levels of Tbet (Tbet dim, Tbet^+^) and 25% expressed high Tbet levels at baseline (Tbet high, Tbet^++^) (Supplementary Figure 4*C* and *D*). Furthermore, Eomes expression on total CD4^+^ T cells in TB-IRIS patients was comparable to non-IRIS controls at all-time points (Supplementary Figure 5*A* and *B*). However, we did observe a modest increase in the frequency of CD4^+^ T cells expressing Eomes between baseline and week 2 (which corresponds to IRIS onset) in TB-IRIS patients (medians: 4.48% vs 7.6%, respectively; *P* = .03). This was not observed in non-IRIS controls (Supplementary Figure 5*C*). Previous studies have reported a higher frequency of both *M avium* and Mtb-specific effector memory CD4 T cells in unmasking and paradoxical TB-IRIS patients compared to non-IRIS patients [24, 34]. Furthermore, a positive correlation between CD4^+^ T-cell memory and Eomes expression is well established [27]. Therefore, it is possible that the increase in Eomes expression observed in total CD4 T cells could be related to an expansion of effector cells.

Finally, Tbet expression on total CD4^+^ T cells was comparable between TB-IRIS and non-IRIS patients at baseline with no significant differences observed longitudinally on ART (data not shown).

**Figure 3.**
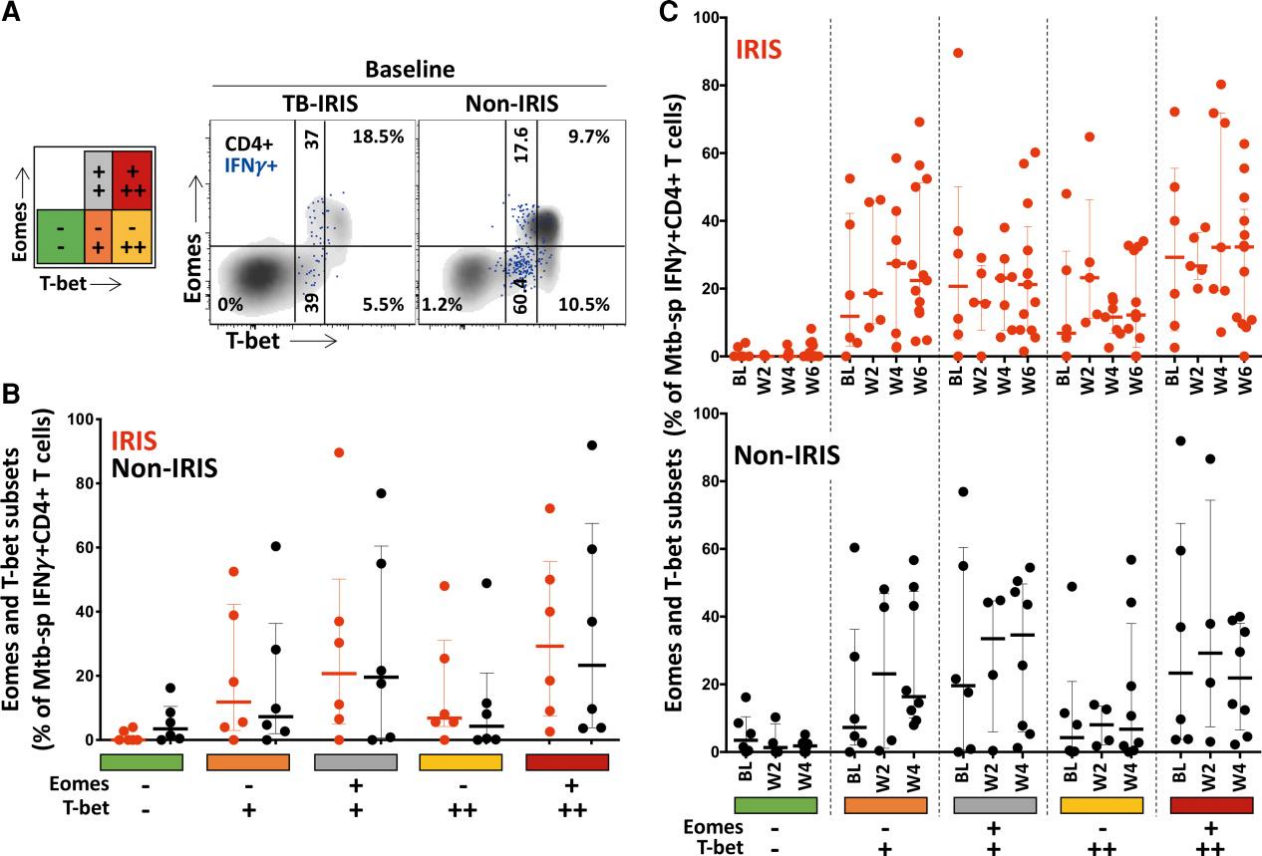
Eomesodermin (Eomes) and Tbet expression profile in *Mycobacterium tuberculosis* (Mtb)-specific IFNγ^+^CD4^+^ T cells in tuberculosis-immune reconstitution inflammatory syndrome (TB-IRIS) and non-IRIS patients. (*A*) Representative flow plot of Eomes and Tbet expression on Mtb-specific IFNγ^+^CD4^+^ T cells (red) and total CD4^+^ T cells (black) in 1 TB-IRIS and 1 non-IRIS patient before antiretroviral therapy [ART] initiation (baseline [BL]). (*B*) Distribution of Mtb-specific IFNγ^+^CD4^+^ T cells among distinct Eomes and Tbet subsets: (Eomes^−^Tbet^−^; Eomes^−^Tbet^+^; Eomes^+^Tbet^+^; Eomes^−^Tbet^++^; Eomes^+^Tbet^++^) in TB-IRIS (red, *n* = 6) and non-IRIS patients (black, *n* = 6) at BL. (*C*) Evolution of Eomes and Tbet profile in Mtb-specific IFNγ^+^CD4^+^ T cells in TB-IRIS from BL, (*n* = 6), through 2 weeks (W2, *n* = 5), 4 weeks (W4, *n* = 7) and 6 weeks (W6, *n* = 13) and non-IRIS patients (black) from BL (*n* = 6), through 2 weeks (W2, *n* = 4) and 4 weeks (W4, *n* = 8) on ART. The Wilcoxon signed-rank test was used for the statistical comparison of paired samples and the Mann-Whitney *U* test was used for unpaired samples. Only statistically significant data with a *P* value of .05 or less are indicated on graphs.

In further analyses, we defined the coexpression of Eomes and Tbet, identifying 5 Eomes/Tbet subsets: Eomes-Tbet-, Eomes-Tbet^+^, Eomes ^+^ Tbet^+^, Eomes-Tbet^++^, and Eomes ^+^ Tbet^++^, as previously described [35] (Figure 3*A*). The distribution of these subpopulations within Mtb-specific IFNγ^+^CD4^+^ T cells was comparable between TB-IRIS and non-IRIS groups before ART initiation (ie, baseline) (Figure 3*B*) and longitudinally on ART (data not shown). No significant changes in the distribution of Eomes and Tbet in Mtb-specific IFNγ^+^CD4^+^ T cells were observed over time within the 2 groups (Figure 3*C*).

However, in total CD4^+^ T cells, there was a significant reduction in Eomes^−^Tbet^−^CD4^+^ T cells between baseline (median: 79.0%; IQR, 21.2–93.1) and week 2 (median: 65.5%; IQR, 15.3–84.4) (*P* = .02) and baseline and week 4 (median: 54.5%; IQR, 21.8–94.5) (*P* = .009) in TB-IRIS patients. These changes were countered by a progressive and significant increase in the proportion of Eomes-Tbet ^+^ and Eomes ^+^ Tbet ^+^ CD4^+^ T cells over the first 6 weeks of ART in TB-IRIS patients. In contrast, no changes over time were observed in the distribution of any of the Eomes/Tbet subsets in non-IRIS patients (Supplementary Figure 6*C*).

### Elevated HLA-DR Expression at the Time of Tuberculosis-Immune Reconstitution Inflammatory Syndrome (IRIS) Onset Compared to Non-IRIS Controls

**Figure 4.**
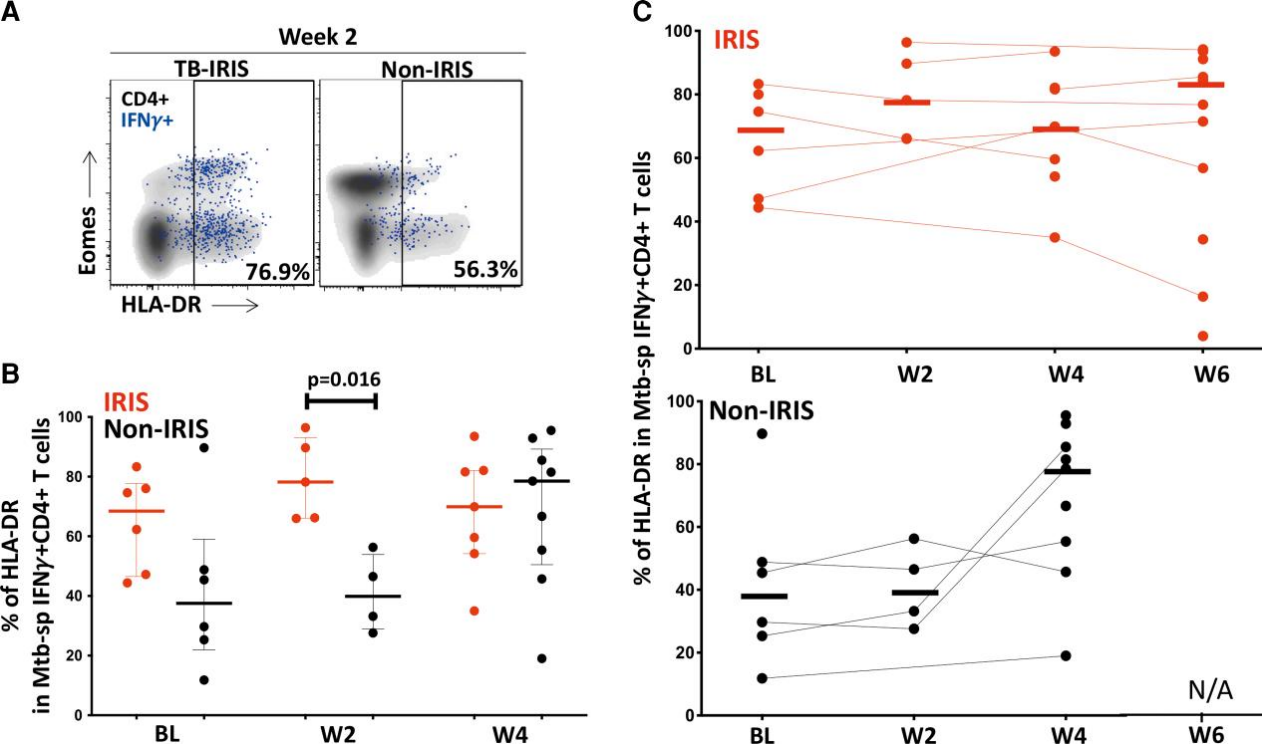
HLA-DR expression on *Mycobacterium tuberculosis* (Mtb)-specific IFNγ^+^CD4^+^ T cells in tuberculosis-immune reconstitution inflammatory syndrome (TB-IRIS) and non-IRIS patients. (*A*) Representative flow plot of HLA-DR expression on Mtb-specific IFNγ^+^CD4^+^ T cells (red) and total CD4^+^ T cells (black) in 1 TB-IRIS and 1 non-IRIS patient at 2 weeks post-antiretroviral therapy (ART) initiation (W2). (*B*) Expression of HLA-DR on Mtb-specific IFNγ^+^CD4^+^ T cells in TB-IRIS (red) from baseline (BL, *n* = 6), through 2 weeks (W2, *n* = 5), 4 weeks (W4, *n* = 7), and 6 weeks (W6, *n* = 13) and non-IRIS patients (black) from baseline (BL, *n* = 6), through 2 weeks (W2, *n* = 4), and 4 weeks (W4, *n* = 8) on ART. (*C*) Frequency of HLA-DR on Mtb-specific IFNγ^+^CD4^+^ T cells from BL to 6 weeks on ART in TB-IRIS and non-IRIS patients. The Wilcoxon signed-rank test was used for the statistical comparison of paired samples, and the Mann-Whitney *U* test was used for unpaired samples. Only statistically significant data with a *P* value of .05 or less are indicated on graphs.

To further characterize the phenotype of Mtb-specific IFNγ^+^CD4^+^ T-cell responses, we compared the activation profile (HLA-DR) and cytotoxic potential (granzyme B) between TB-IRIS patients and non-IRIS controls. We observed a trend towards high pre-ART HLA-DR expression (*P* = .18) in TB-IRIS compared to non-IRIS patients. Responses were characterized by a significantly higher expression of HLA-DR in TB-IRIS compared to non-IRIS patients at 2 weeks on ART (median: 79.3% [IQR, 66–96] and 40.9% [IQR, 27–56], respectively; *P* = .016) (Figure 4*A*and*B*). No significant changes over time were observed when data were analyzed longitudinally for both groups, respectively (Figure 4*C*). However, we observed an increase in HLA-DR expression in total CD4^+^ T cells in TB-IRIS patients between baseline and week 2 (*P* = .002) and week 4 (*P* = .0005) and non-IRIS patients between baseline and week 4 (*P* = .0098) (Supplementary Figure 7).

### Elevated HLA-DR and Granzyme B Expression in *Mycobacterium tuberculosis*-Specific CD4 T Cells Coexpressing Eomesodermin and Tbet in Patients With Tuberculosis-Immune Reconstitution Inflammatory Syndrome (IRIS) Compared to Non-IRIS Controls

**Figure 5.**
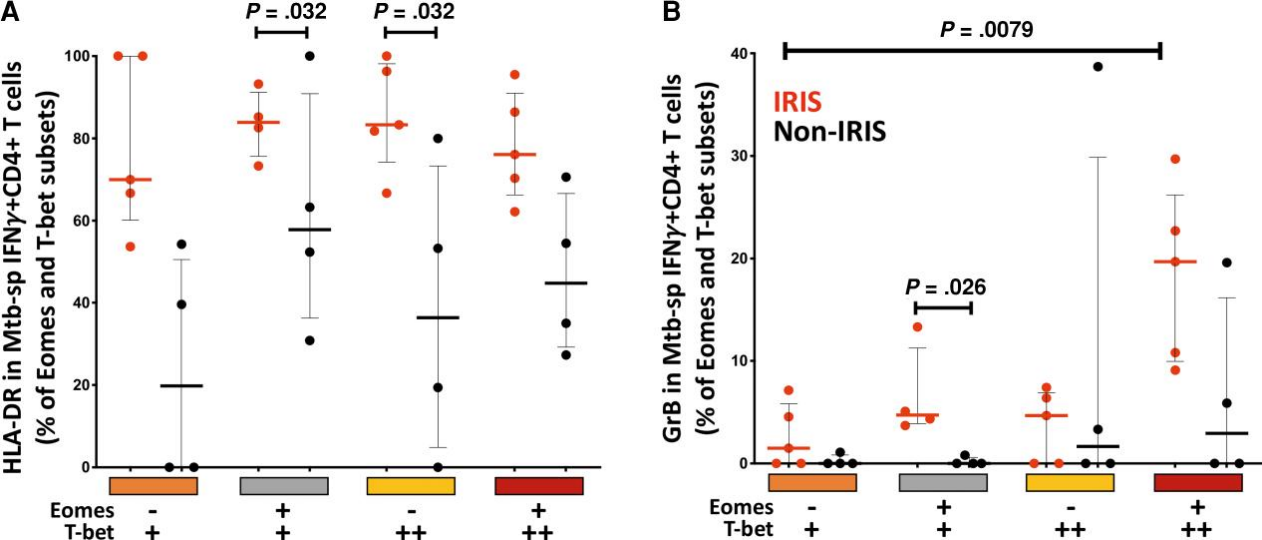
Expression of HLA-DR and granzyme B on Eomesodermin (Eomes) and Tbet-expressing subsets of *Mycobacterium tuberculosis* (Mtb)-specific IFNγ^+^CD4^+^ T cells 2 weeks on antiretroviral therapy (ART). (*A*) Expression of HLA-DR and (*B*) granzyme B on Eomes and Tbet subsets (Eomes^−^, Tbet^+^, Eomes^+^, Tbet^+^, Eomes^−^, Tbet^++^ and Eomes^+^, Tbet^++^) of Mtb-specific IFNγ^+^CD4^+^ T cells in TB-IRIS (red, *n* = 5), and non-IRIS patients (black, *n* = 4), 2 weeks on ART. The Mann-Whitney *U* test was used for statistical comparison of unpaired samples. Only statistically significant data with a *P* value of .05 or less are indicated on graphs.

Finally, we investigated the activation and cytotoxic potential of Mtb-specific IFNγ^+^CD4^+^ T cells in relation to their transcription factor profile at the time of IRIS onset (week 2). Although HLA-DR expression was comparable across the different Eomes and Tbet subsets in both groups, HLA-DR expression was higher in TB-IRIS compared to non-IRIS patients in specific Eomes/Tbet subsets, including Eomes ^+^ Tbet ^+^ (median: 83.9% vs 57.9%, respectively; *P* = .032) and Eomes- Tbet^++^ (median: 83.3% vs 36.4%, respectively; *P* = .032) (Figure 5*A*).

It is notable that no differences in granzyme B expression were observed in IFNγ^+^CD4 T cells between the 2 groups in cross-sectional comparisons (Supplementary Figure 8). However, granzyme B expression was significantly higher in Eomes ^+^ Tbet ^+^ Mtb-specific IFNγ^+^CD4^+^ T cells in patients with TB-IRIS compared to non-IRIS controls at week 2 on ART (median: 4.7% vs 0%, respectively; *P* = .026). There was also a trend towards higher granzyme B expression in the Eomes ^+^ Tbet^++^ Mtb-specific IFNγ^+^CD4 ^+^ subset in patients with TB-IRIS compared to non-IRIS controls at week 2 (median: 19.7% vs 2.9%, respectively; *P* = .063) (Figure 5*B*). This trend was not observed at other time points (data not shown).

## DISCUSSION

Hsu et al [26] recently reported that in HIV-1 and *M avium* coinfected patients, *M avium*-specific IFNγ^+^CD4^+^ T cells were characterized by higher expression of Eomes than Tbet at IRIS onset, suggesting potential involvement of Eomes in mycobacterial IRIS pathogenesis. Although the functional role of Eomes is well established in CD8 T cells [27, 28], its role in CD4 T cells is less clear. Some reports implicate its expression in the pathogenesis of chronic inflammatory disorders [36–38], whereas others suggest a regulatory role in T cells [39]. Therefore, to define whether aberrant expression of transcription factors in CD4 T cell associate with the development of IRIS, we investigated the role of Eomes and Tbet in an experimentally induced MAC-IRIS mouse model and compared the phenotype of Mtb-specific IFNγ^+^CD4^+^ T cells between HIV-associated TB patients who developed TB-IRIS and those who did not.

The MAC-IRIS mouse model showed that mimicking T-cell reconstitution using Eomes KO CD4 T cells led to enhanced mice survival compared to WT, supporting the hypothesis that Eomes expression in CD4 T cells could play a role in IRIS pathogenesis [26]. However, although we demonstrated that Mtb-specific IFNγ^+^CD4^+^ T cells from TB-IRIS patients expressed high Eomes levels (∼50%) that are comparable to those reported by Hsu et al [26], we did not observe any difference in Eomes expression between TB-IRIS and non-IRIS patients. Several reasons could explain the disparity between our findings and the findings of Hsu et al [26]. One notable difference is that the HIV-infected patients studied in the Hsu et al [26] study had unmasking MAC-IRIS (10 of 13) and were compared with HIV-uninfected patients with MAC infection. In this study, all patients with IRIS had paradoxical TB-IRIS, and the control group was constituted of HIV-infected TB patients who did not develop TB IRIS after initiating ART. It is possible that the immunopathology of unmasking versus paradoxical IRIS is regulated by different mechanisms. It is also possible that differences in the underlying pathogen (MAC versus Mtb) may have contributed, but we think that this is unlikely given the considerable overlap in the inflammatory features and tissue pathology of IRIS related to these pathogens. Moreover, HIV infection itself may alter the expression of lineage defining transcription factor, as previously described [40]. Hence, it would be of interest to define whether differential Eomes expression in unmasking MAC-IRIS patients is still observed when compared to HIV-infected matched control patients with MAC initiating ART. Nevertheless, because limited samples were included in both studies, larger cohorts are necessary to define the potential role of Eomes in unmasking and paradoxical IRIS.

The expression profile of Tbet in CD4^+^ T cells in this cohort mirrors that described by Knox et al [41], where 3 distinct populations were discernible. Most Mtb-specific IFNγ^+^CD4^+^ T cells expressed Tbet with ∼65% being Tbet dim and ∼25% Tbet bright. Moreover, we found no significant differences in the coexpression profile of Eomes and Tbet in Mtb-specific IFNγ^+^CD4^+^ T cells at TB-IRIS onset or at other time points between the 2 clinical groups. However, the distribution of Eomes and Tbet subsets in total CD4^+^ T cells were altered on ART with increasing expression of both Tbet ^+^ and Eomes/Tbet coexpressing CD4^+^ T cells in TB-IRIS patients on ART. Further studies are needed to confirm these observations and define their relevance.

In this cohort, TB-IRIS patients had significantly lower blood CD4 T-cell counts compared to non-IRIS patients at baseline, as previously described [8, 42], and we observed a significant expansion in the frequency of Mtb-specific IFNγ^+^CD4^+^ T cells 2 weeks after the initiation of ART. In a recent study, Vignesh et al [42] described elevated pre-ART frequencies of Mtb-specific CD4 T-cell responses that further expanded in TB-IRIS patients at disease onset. We did not observe such differences at baseline in this or previous studies [23]. Clinical differences between the cohorts might account for these discrepancies.

Several studies have demonstrated that TB-IRIS is characterized by an increase in mycobacteria-specific CD4 T-cell responses at disease onset [22, 42–45]. However, increased mycobacterial-specific CD4 T-cell frequencies after ART is not systematically observed in all TB-IRIS patients, and pathogen-specific CD4 T-cell expansion can also be observed in some non-IRIS patients [23]. This suggests that Mtb-specific CD4 T-cell reconstitution upon ART is not the only mechanism involved in TB-IRIS pathogenesis. The latest evidence suggests that paradoxical TB-IRIS is also partly driven by *M tuberculosis*-primed innate immune responses, including inflammasome activation, being activated after ART initiation [9]. *Mycobacterium tuberculosis*-specific CD4 T cells may provide activation signals to the already primed innate immune cells resulting in a dysregulated inflammatory response.

To further elucidate the contribution of Mtb-specific IFNγ^+^CD4^+^ T cells in TB-IRIS pathology, we characterized their phenotype in TB-IRIS patients. We demonstrated that Mtb-specific IFNγ^+^CD4 T cells of TB-IRIS patients had elevated HLA-DR expression before the initiation of ART, and this was significantly upregulated in TB-IRIS patients at week 2 on ART compared to non-IRIS patients. Likewise, others have demonstrated that Mtb-specific CD4 T cells are activated [24] and polyfunctional [25, 43], compared to non-IRIS controls at IRIS onset.

Consistent with our previous findings [23], we did not observe any significant differences in the expression of HLA-DR in total CD4^+^ T cells between the 2 clinical groups over time in a cross-section analysis. However, similar to Antonelli et al [24], we observed increased HLA-DR expression in total CD4^+^ T cells of TB-IRIS patients from baseline to week 2 and 4. Similar observations were reported by Haridas et al [46] at the time of IRIS onset.

Finally, granzyme B expression was enriched in Eomes/Tbet coexpressing Mtb-specific IFNγ^+^CD4^+^ T cells at 2 weeks on ART in TB-IRIS patients. Although this represents a modest proportion of Eomes ^+^ Tbet ^+^ cells, this is consistent with mouse data from experimental autoimmune encephalitis showing the capacity of Eomes ^+^ IFNγ^+^CD4 T cells to acquire cytotoxic attributes [36]. Moreover, our group has previously shown TB-IRIS to be associated with increased transcript abundance and secretion of granzyme B by PBMC of TB-IRIS patients at week 2 on ART [47].

### Study Limitations

There were several limitations to this study. The number of samples analyzed was limited, and, consequently, larger cohort studies are needed to verify these findings. We assessed responses in peripheral blood when clinical manifestations are often localized in tissue. Furthermore, the cell viability of the analyzed samples was highly variable. To mitigate this potential technical issue, precautions were taken to include only live cells in our analysis, through stringent application of gates, excluding dead cells. Finally, several patients with severe disease received corticosteroids before or while on ART, the main indications being neurological TB at TB diagnosis or the development of TB-IRIS after initiating ART. However, our previous findings suggest that corticosteroid treatment does not have a significant impact on ex vivo T-cell functional responses in TB-IRIS patients [33].

## CONCLUSIONS

In conclusion, although the mouse model data suggested that CD4 T-cell expression of Eomes promotes IRIS, there were no differences in the expression of Eomes or Tbet transcription factor in Mtb-specific IFNγ^+^ CD4 T cells between patients who developed TB-IRIS and non-IRIS controls. We found that TB-IRIS was associated with an increase of Mtb-specific CD4 T cells at onset. Moreover, increased expression of markers of immune activation and cytotoxicity in Mtb-specific CD4 T-cell subsets in TB-IRIS patients suggests these cells may contribute to pathogenesis of TB-IRIS. Improved understanding of the pathophysiology of IRIS should enable the development of new diagnostic tools and better targeted treatments.

## Supplementary Material

ofac546_Supplementary_Data

## Supplementary Data

Supplementary materials are available at *Open Forum Infectious Diseases* online. Consisting of data provided by the authors to benefit the reader, the posted materials are not copyedited and are the sole responsibility of the authors, so questions or comments should be addressed to the corresponding author.
