# Supplementary material for: Phenotypic Profile of Mycobacterium tuberculosis-Specific CD4 T-Cell Responses in People With Advanced Human Immunodeficiency Virus Who Develop Tuberculosis-Associated Immune Reconstitution Inflammatory Syndrome
Source: Open Forum Infect Dis. 2022 Oct 17;10(1):ofac546. doi: 10.1093/ofid/ofac546 (PMC9879713; doi:10.1093/ofid/ofac546)
Supplement: ofac546_Supplementary_Data [file ofac546_supplementary_data.zip › Moseki_R_Supplementary_Figures_OFID_Version24 copy.pdf]

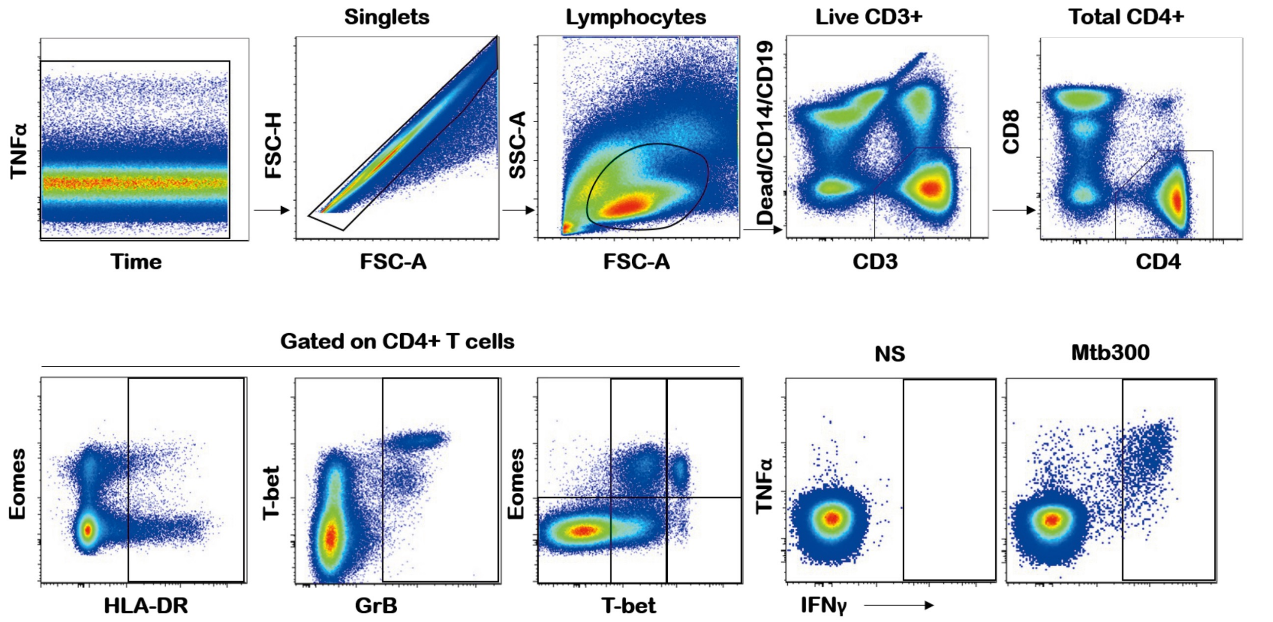

Supplementary Figure 1. Representative gating strategy for the phenotypic characterization of IFNγ+CD4+ T cells. To phenotype Mtb-specific IFNγ+CD4+ T cell responses, we gated on singlets (FSC-H vs FSC-A), lymphocytes (SSC-A vs FSC-A), live CD3+ cells (dead cells vs live CD3+) and on total CD4+ T cells.

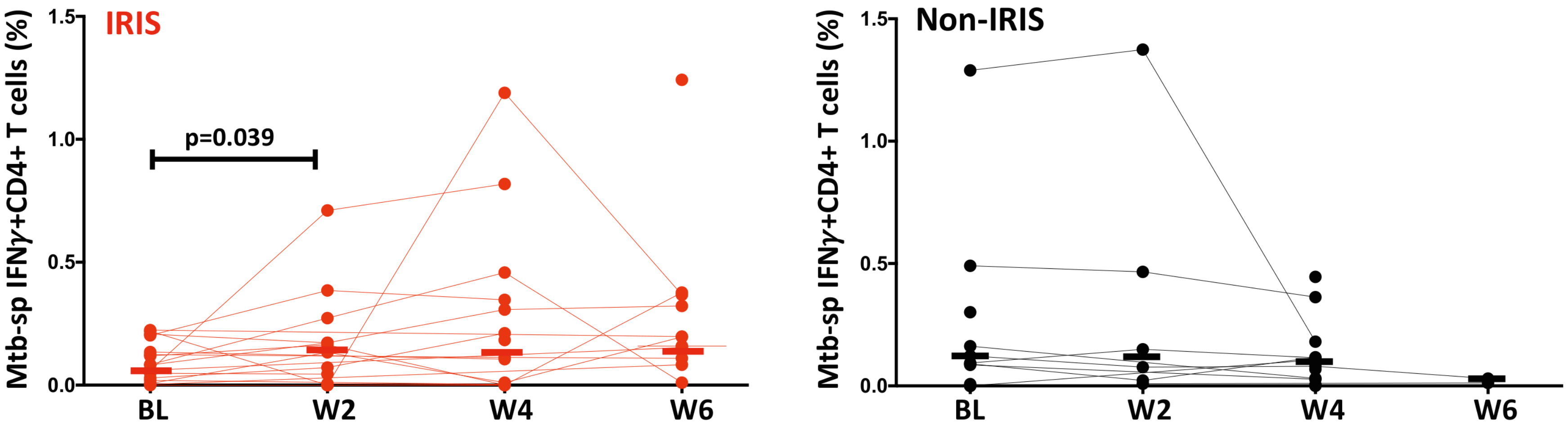

Supplementary Figure 2. Frequencies of IFNγ producing CD4+ T cells in response to Mtb peptide pool (Mtb300) stimulation in TB-IRIS (red) at baseline (BL, n= 16), 2 weeks (W2, n= 9), 4 weeks (W4, n= 10) and 6 weeks (W6, n=12) and non-IRIS (black) at BL= 11, W2, n= 4, W4, n= 8 and W6, n= 1 after ART initiation. The Wilcoxon ranked test was used for all statistical comparisons. Only statistically significant data with a p value of 0.05 or less are indicated on graphs.

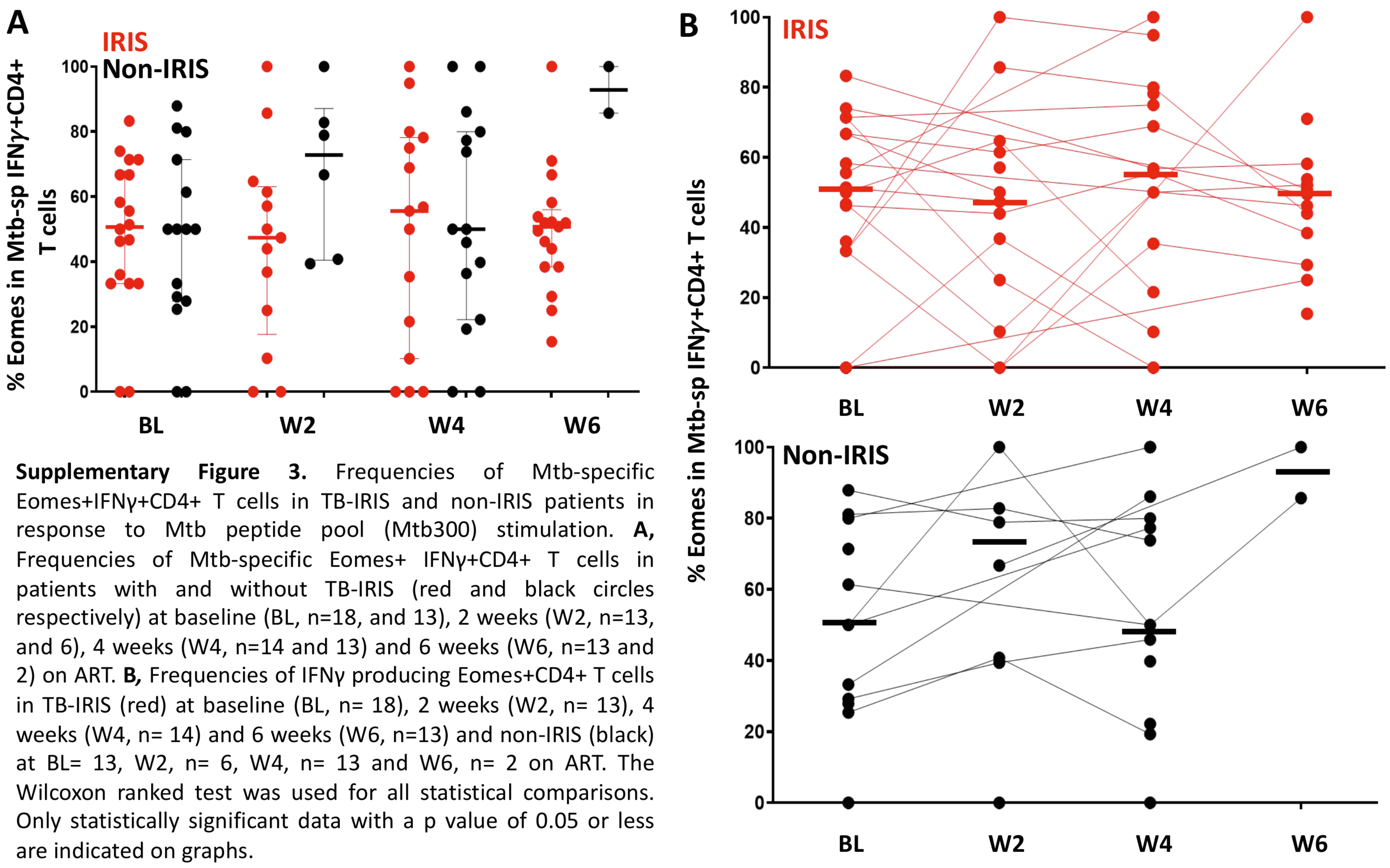

Supplementary Figure 3. Frequencies of Mtb-specific Eomes+IFNγ+CD4+ T cells in TB-IRIS and non-IRIS patients in response to Mtb peptide pool (Mtb300) stimulation. **A,** Frequencies of Mtb-specific Eomes+ IFNγ+CD4+ T cells in patients with and without TB-IRIS (red and black circles respectively) at baseline (BL, n=18, and 13), 2 weeks (W2, n=13, and 6), 4 weeks (W4, n=14 and 13) and 6 weeks (W6, n=13 and 2) on ART. **B,** Frequencies of IFNγ producing Eomes+CD4+ T cells in TB-IRIS (red) at baseline (BL, n= 18), 2 weeks (W2, n= 13), 4 weeks (W4, n= 14) and 6 weeks (W6, n=13) and non-IRIS (black) at BL= 13, W2, n= 6, W4, n= 13 and W6, n= 2 on ART. The Wilcoxon ranked test was used for all statistical comparisons. Only statistically significant data with a p value of 0.05 or less are indicated on graphs.

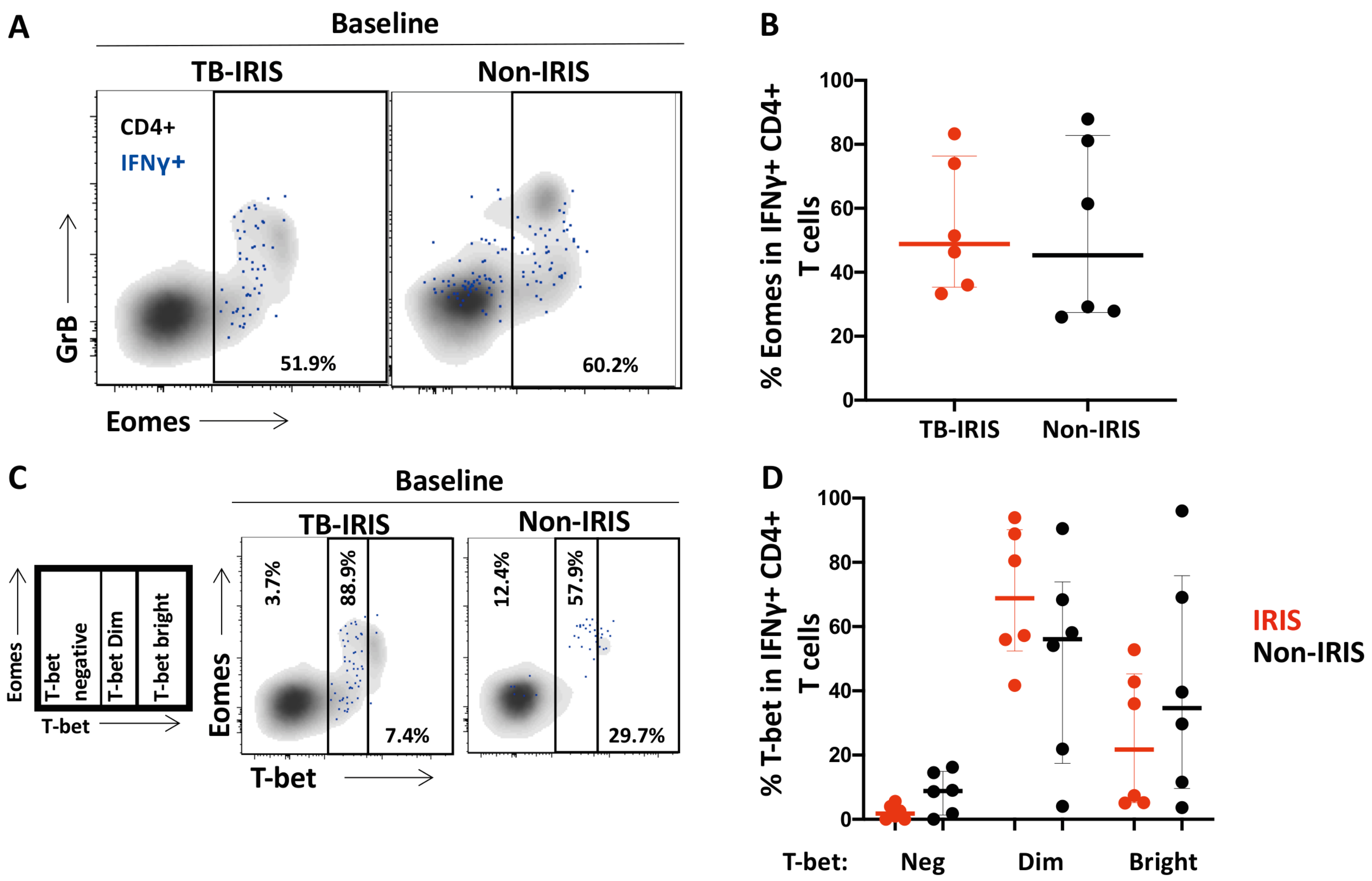

Supplementary Figure 4. Eomes and T-bet expression on Mtb-specific IFNγ+CD4+ T cells in patients with and without TB-IRIS prior to initiation of antiretroviral therapy (ART) (BL). A, Representative flow plot of the expression of Eomes on Mtb-specific IFNγ+CD4+ T cells (red) and total CD4+ T cells (black) in one TB-IRIS and one non-IRIS patient at BL. **B,** Summary plot of Eomes expression in Mtb-specific IFNγ+CD4+ T cells between TB-IRIS (n= 6) and non-IRIS patients (n= 6) at BL. **C,** Representative flow plot of the expression of differentiated T-bet subpopulations on Mtb-specific IFNγ+CD4+ T cells (red) and total CD4+ T cells (black) in one TB-IRIS and one non-IRIS patient at baseline. **D,** Summary plot of the T-bet expression in Mtb-specific IFNγ+CD4+ T cells between TB-IRIS (n= 6) and non-IRIS patients (n= 6) at BL. The Wilcoxon ranked test was used for all statistical comparisons. Only statistically significant data with a p value of 0.05 or less are indicated on graphs.

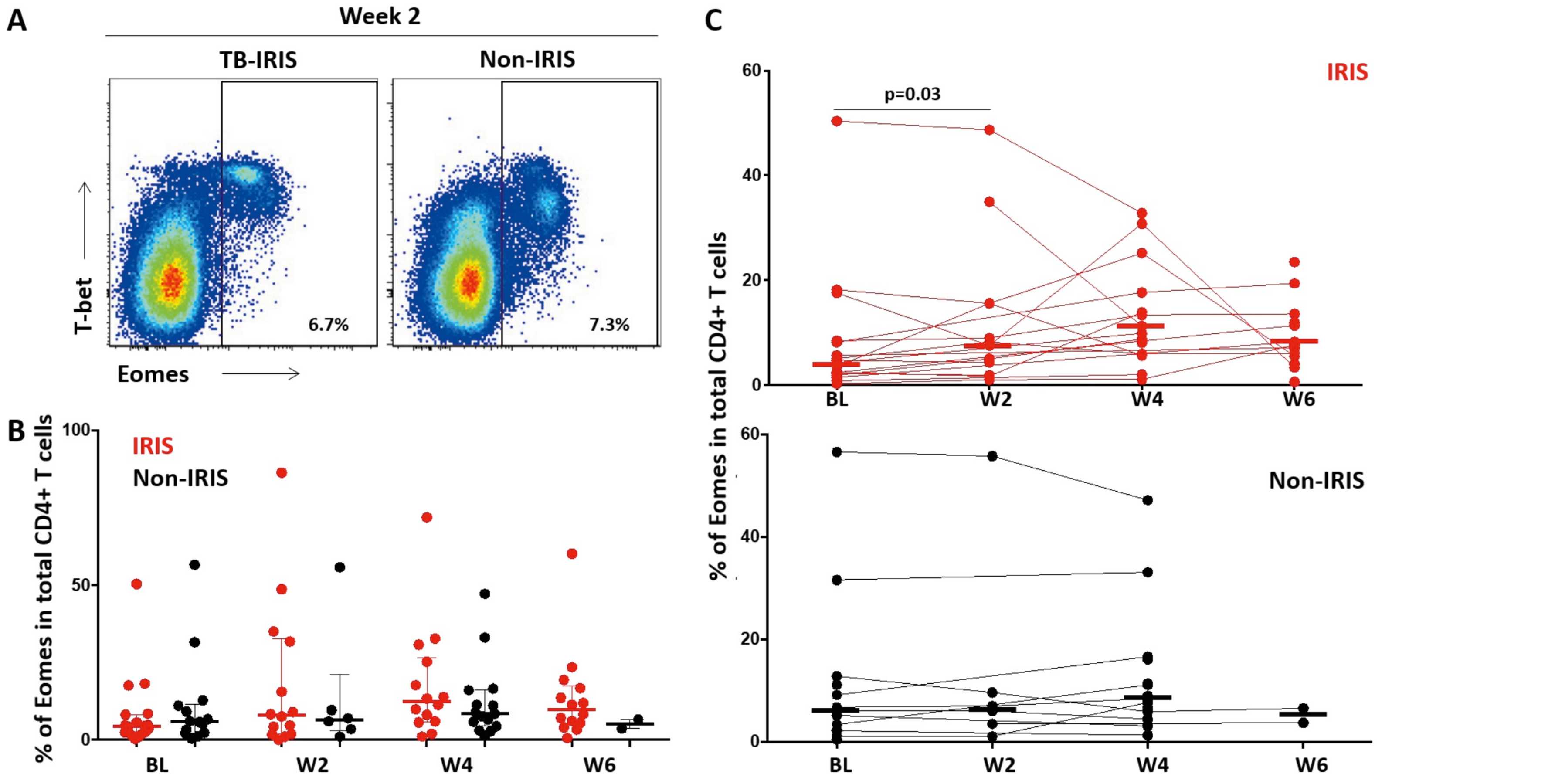

Supplementary Figure 5. Eomes expression in total CD4+ T cells in patients with and without TB-IRIS. **A,** Representative flow plot of Eomes expression in one patient with TB-IRIS and one non-IRIS patient in total CD4+ T cells two weeks post ART initiation. **B,** Cross sectional analyses of Eomes expression in total CD4+ T cells in patients with and without TB-IRIS at baseline (BL, n=18, and 13, respectively), 2 weeks (W2, n=13, and 6), 4 weeks (W4, n=14 and 13) and 6 weeks (W6, n=13 and 2) post-ART. **C,** Longitudinal analyses of the expression of Eomes in total CD4+ T cells in patients with TB-IRIS (top panel) and non-IRIS controls (bottom panel) from BL to 6 weeks post ART. The Wilcoxon ranked test was used for all statistical comparisons. Only statistically significant data with a p value of 0.05 or less are indicated on graphs.

Supplementary Fig. 6

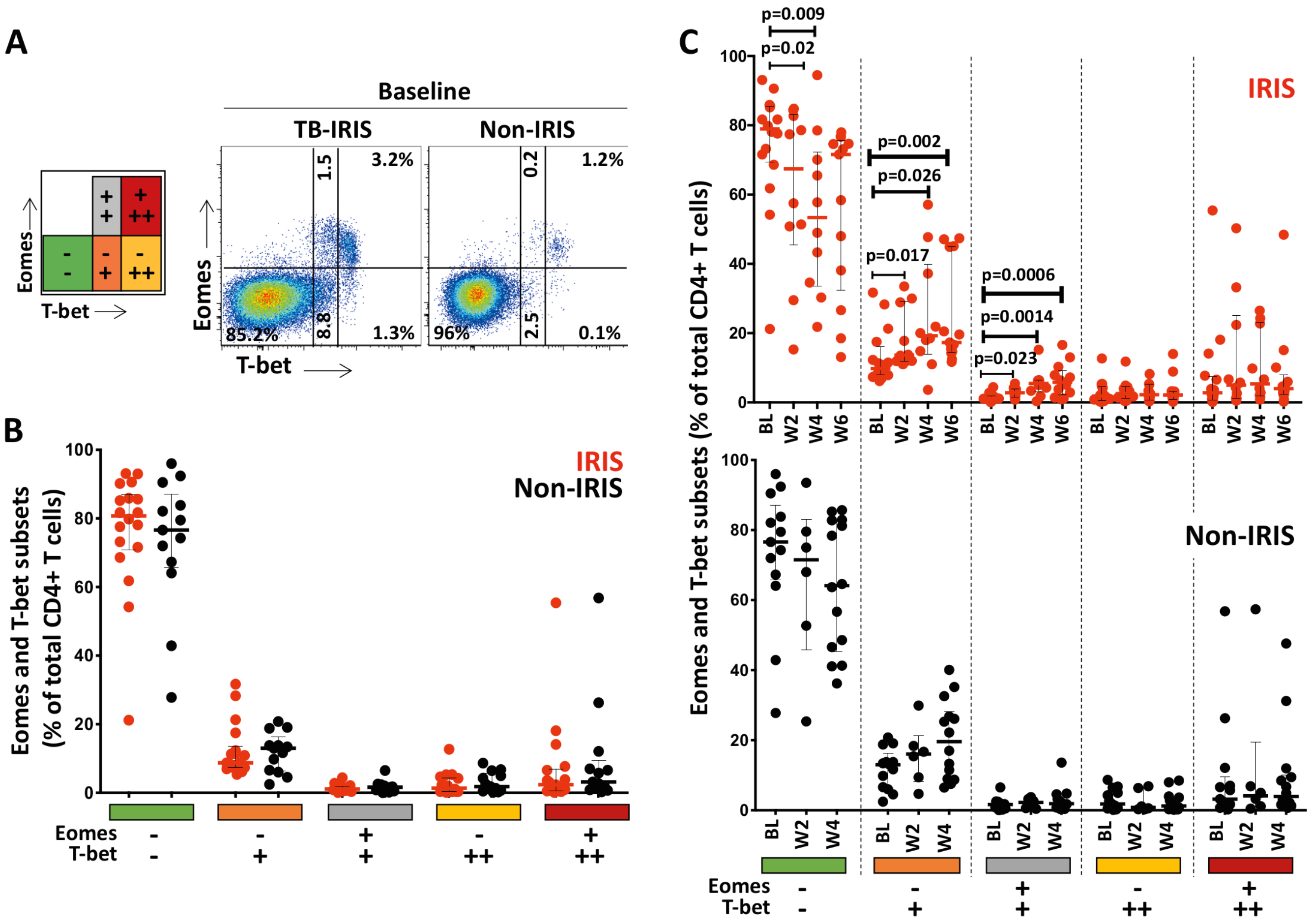

Supplementary Figure 6. Eomes and T-bet co-expression in total CD4+ T cells in patients with and without TB-IRIS. **A,** Representative flow plot of Eomes and T-bet co-expression in one patient with TB-IRIS and one non-IRIS patient in total CD4+ T cells two weeks on ART initiation. **B,** Cross sectional analyses of Eomes and T-bet co-expression in total CD4+ T cells in patients with and without TB-IRIS at baseline (BL, n=18, and 13, respectively), 2 weeks (W2, n=13, and 6), 4 weeks (W4, n=14 and 13) and 6 weeks (W6, n=13 and 2) on ART. **C,** Longitudinal analyses of the co-expression of Eomes and T-bet in total CD4+ T cells in patients with TB-IRIS (top panel) and non-IRIS controls (bottom panel) from BL to 6 weeks on ART. The Wilcoxon ranked test was used for all statistical comparisons. Only statistically significant data with a p value of 0.05 or less are indicated on graphs.

Supplementary Fig. 7

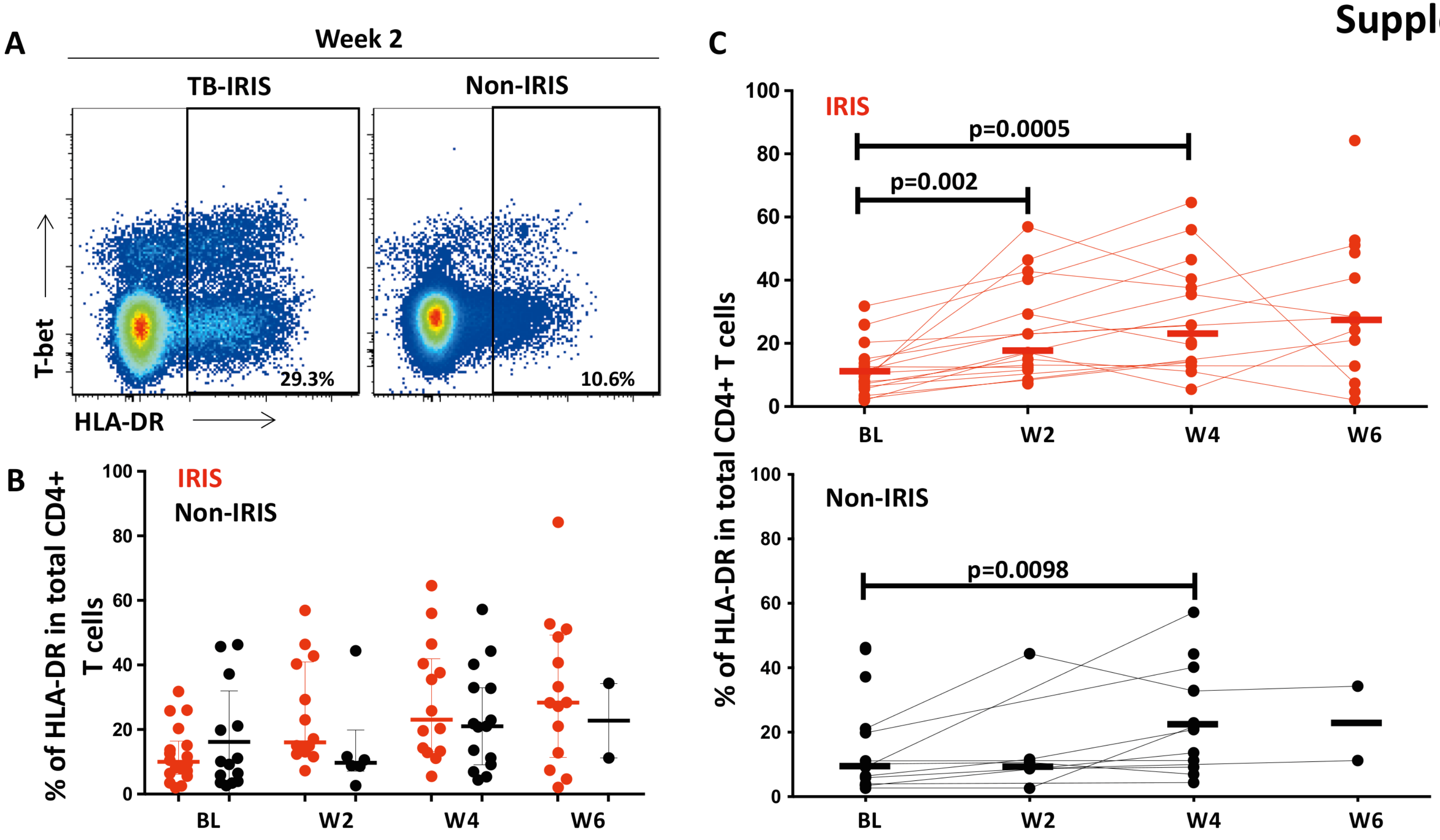

Supplementary Figure 7. HLA-DR expression in total CD4+ T cells in patients with and without TB-IRIS. **A,** Representative flow plot of HLA-DR expression in one patient with TB-IRIS and one non-IRIS patient in total CD4+ T cells two weeks post ART initiation. **B,** Cross sectional analyses of HLA-DR expression in total CD4+ T cells in patients with and without TB-IRIS at baseline (BL, n=18, and 13, respectively), 2 weeks (W2, n=13, and 6), 4 weeks (W4, n=14 and 13) and 6 weeks (W6, n=16 and 2) post-ART. **C,** Longitudinal analyses of the expression of HLA-DR in total CD4+ T cells in patients with TB-IRIS from BL, n= 18, W2, n= 13,W4, n= 14, W6, n= 13 and non-IRIS controls, BL, n= 13, W2, n= 6, W4, n= 13 and W6, n= 2 post ART. The Wilcoxon ranked test was used for all statistical comparisons. Only statistically significant data with a p value of 0.05 or less are indicated on graphs.

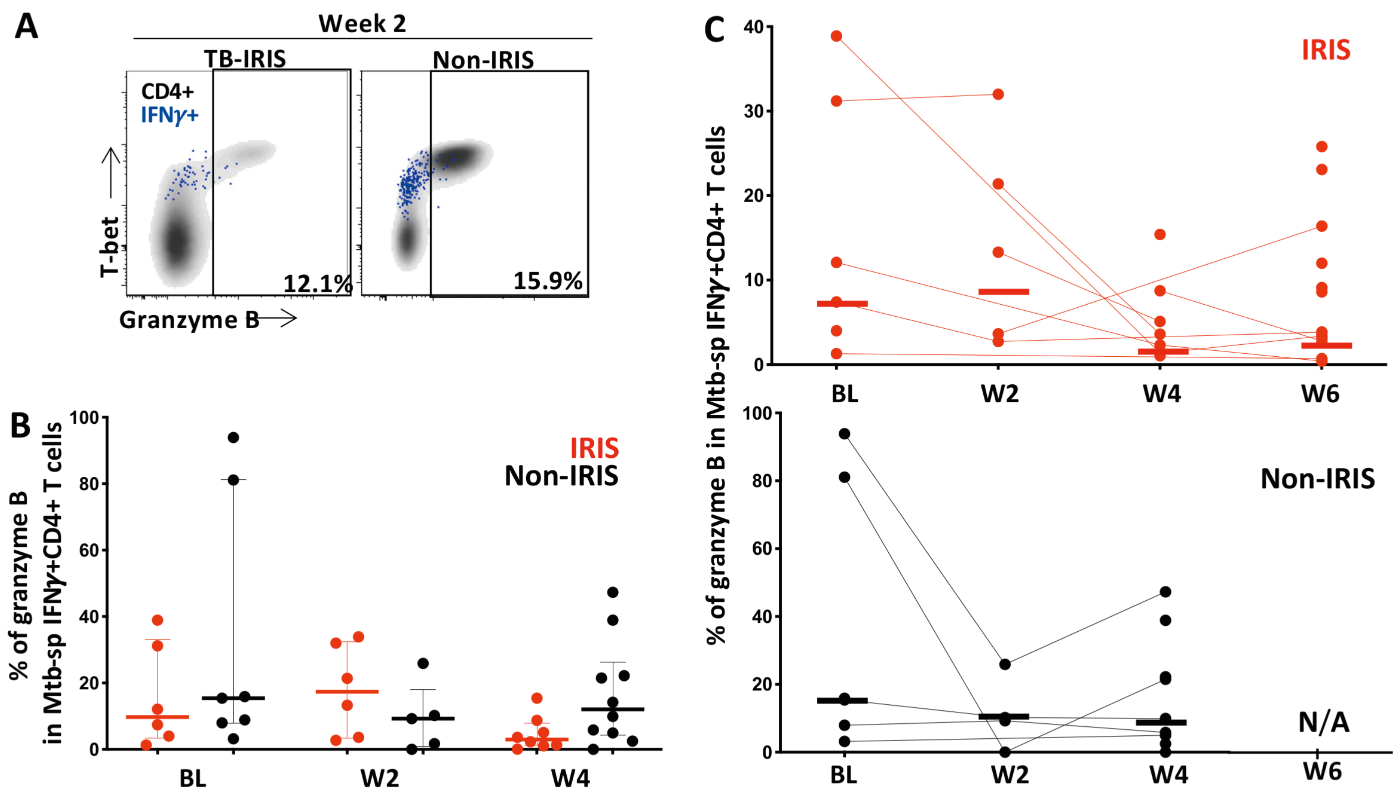

Supplementary Figure 8. **Granzyme B expression in Mtb-specific IFNγ+CD4+ T cells in TB-IRIS and non-IRIS patients**. **A,** Representative flow plot of granzyme B expression in Mtb-specific IFNγ+CD4+ T cells (red) and total CD4+ T cells (gray) in one TB-IRIS and one non-IRIS patient prior to ART initiation (at Baseline, BL). **B,** Expression of granzyme B in Mtb-specific IFNγ+CD4+ T cells in TB-IRIS (red) at baseline (BL, n= 6), 2 weeks (W2, n= 5), 4 weeks (W4, n= 7) and 6 weeks (W6, n= 13) and non-IRIS patients (black) at baseline (BL, n= 6), 2 weeks (W2, n= 4), and 4 weeks (W4, n= 8) post-ART. **C,** Expression of granzyme B in Mtb-specific IFNγ+CD4+ T cells from Baseline to 6 weeks post ART in TB-IRIS and non-IRIS patients. The Wilcoxon ranked test was used for all statistical comparisons. Only statistically significant data with a p value of 0.05 or less are indicated on graphs.
