# Supplementary material for: Phenotypic Profile of Mycobacterium tuberculosis-Specific CD4 T-Cell Responses in People With Advanced Human Immunodeficiency Virus Who Develop Tuberculosis-Associated Immune Reconstitution Inflammatory Syndrome
Source: Open Forum Infect Dis. 2022 Oct 17;10(1):ofac546. doi: 10.1093/ofid/ofac546 (PMC9879713; doi:10.1093/ofid/ofac546)
Supplement: ofac546_Supplementary_Data [file ofac546_supplementary_data.zip › Supp Table 1_RM.pdf]

Supplementary Table 1

Patient Identifier	ART regimen	Duration ART Start to TB-IRIS (days)	TB-IRIS system involvement	Steroids to treat TB-IRIS	Duration from TB-IRIS onset to starting steroids (days)
**IR 4011-3**	D4T, 3TC, EFZ	6	Pulmonary, nodal, effusion and abdominal	Yes	9
**IR 4035-1**	AZT, 3TC, EFZ	42	Nodal, abdominal	No	-
**IR 4074-8**	TDF, 3TC, EFZ	10	Pulmonary, nodal	Yes	5
**IR 4071-5**	TDF, 3TC, EFZ	8	Pulmonary, effusion, abdominal	No	-
**IR 4075-9**	D4T, 3TC, EFZ	12	Abdominal	Yes	na
**IR 4076-0**	TDF, 3TC, EFZ	9	Nodal	No	-
**IR 4081-7**	TDF, 3TC, EFZ	10	Abdominal	No	-
**IR 4108-1**	TDF, 3TC, EFZ	9	Pulmonary, abdominal	No	-
**IR 4018-0**	D4T, 3TC, EFZ	13	Pulmonary	Yes	4
**IR 4020-4**	D4T, 3TC, EFZ	30	Nodal	Yes	13
**IR 4047-5**	D4T, 3TC, EFZ	8	Pulmonary, abdominal	Yes	5
**IR 4110-5**	D4T, 3TC, EFZ	4	Pulmonary, abdominal	No	-
**IR 4052-2**	TDF, 3TC, EFZ	13	Pulmonary, abdominal	No	-
**IR 4078-2**	TDF, 3TC, EFZ	26	Neurological, pulmonary, abdominal	Yes	20
**IR 4096-4**	TDF, 3TC, EFZ	14	Pulmonary, abdominal	No	-
**IR 4115-0**	AZT, 3TC, EFZ	26	Pulmonary	Yes	16
**IR 4010-2**	D4T, 3TC, EFZ	7	Nodal, abdominal	Yes	56
**IR 4015-7**	D4T, 3TC, EFZ	15	Pulmonary, abdominal	Yes	20
**IR 4021-8**	D4T, 3TC, EFZ	5	Pulmonary	Yes	2
**IR 4036-2**	D4T, 3TC, EFZ	7	Nodal, neurological	Yes	1
**IR 4080-6**	TDF, 3TC, EFZ	11	Neurological	Yes	na
**IR 4085-1**	TDF, 3TC, EFZ	14	Articular	Yes	na
**IR 4088-4**	TDF, 3TC, EFZ	49	Neurological	Yes	9
**IR 4095-3**	TDF, 3TC, EFZ	12	Pulmonary, abdominal	Yes	3
**IR 4111-6**	TDF, 3TC, EFZ	8	Abdominal	Yes	8

Supplementary Table 1. Individual patient duration to first TB-IRIS episode, symptom manifestation and steroid management. Different patients were on different combination of ART regimen including Stavudine (D4T), Lamivudine (3TC), Efavirenz (EFZ), Zidovudine (AZT) and Tenofovir (TDF).
